# A Brazilian Inter-Hospital Candidemia Outbreak Caused by Fluconazole-Resistant *Candida parapsilosis* in the COVID-19 Era

**DOI:** 10.3390/jof8020100

**Published:** 2022-01-20

**Authors:** Danilo Y. Thomaz, Gilda M. B. Del Negro, Leidiane B. Ribeiro, Mirian da Silva, Gabrielle O. M. H. Carvalho, Carlos H. Camargo, João N. de Almeida, Adriana L. Motta, Rinaldo F. Siciliano, Odeli N. E. Sejas, Flávia Rossi, Edson Abdala, Tânia M. V. Strabelli, Gil Benard

**Affiliations:** 1 Laboratory of Medical Mycology (LIM-53), Instituto de Medicina Tropical e Hospital das Clínicas da Faculdade de Medicina da Universidade de São Paulo, Sao Paulo 05403-000, Brazil; dan.yamamoto.thz@usp.br (D.Y.T.); gildadelnegro@gmail.com (G.M.B.D.N.); leidiane.lr@hotmail.com (L.B.R.); mirian.07silva@gmail.com (M.d.S.); gabriellehaddad@usp.br (G.O.M.H.C.); 2 Bacteriology Center, Instituto Adolfo Lutz, Sao Paulo 01246-000, Brazil; carlos.pqc@gmail.com; 3 Central Laboratory Division (LIM-03), Hospital das Clínicas da Faculdade de Medicina da Universidade de São Paulo, Sao Paulo 05403-010, Brazil; jnaj99@gmail.com (J.N.d.A.J.); adriana.motta@hc.fm.usp.br (A.L.M.); flaviarossi61@gmail.com (F.R.); 4 Infection Control Team, Heart Institute (InCor), Hospital das Clínicas da Faculdade de Medicina da Universidade de São Paulo, Sao Paulo 05403-000, Brazil; rinaldo.siciliano@c.fm.usp.br (R.F.S.); tania.s@hc.fm.usp.br (T.M.V.S.); 5 Cancer Institute of São Paulo State, Hospital das Clínicas da Faculdade de Medicina da Universidade de São Paulo, Sao Paulo 01246-000, Brazil; odeli.sejas@hc.fm.usp.br (O.N.E.S.); edson.abdala@hc.fm.usp.br (E.A.)

**Keywords:** inter-hospital transmission, clonal outbreak, candidemia, *Candida parapsilosis*, microsatellite typing, antifungal agents, azole-resistant, drug resistance mechanisms, *ERG11* mutations, COVID-19 pandemic

## Abstract

Horizontal transmission of fluconazole-resistant *Candida parapsilosis* (FRCP) through healthcare workers’ hands has contributed to the occurrence of candidemia outbreaks worldwide. Since the first COVID-19 case in Brazil was detected in early 2020, hospitals have reinforced hand hygiene and disinfection practices to minimize SARS-CoV-2 contamination. However, a Brazilian cardiology center, which shares ICU patients with a cancer center under a FRCP outbreak since 2019, reported an increased FRCP candidemia incidence in May 2020. Therefore, the purpose of this study was to investigate an inter-hospital candidemia outbreak caused by FRCP isolates during the first year of the COVID-19 pandemic in Brazil. *C. parapsilosis* bloodstream isolates obtained from the cancer (*n* = 35) and cardiology (*n* = 30) centers in 2020 were submitted to microsatellite genotyping and fluconazole susceptibility testing. The *ERG11* gene of all isolates from the cardiology center was sequenced and compared to the corresponding sequences of the FRCP genotype responsible for the cancer center outbreak in 2019. Unprecedentedly, most of the FRCP isolates from the cardiology center presented the same genetic profile and Erg11-Y132F mutation detected in the strain that has been causing the persistent outbreak in the cancer center, highlighting the uninterrupted horizontal transmission of clonal isolates in our hospitals during the COVID-19 pandemic.

## 1. Introduction

Candidemia, the most frequent clinical manifestation of invasive candidiasis, presents a high risk of morbidity and mortality, especially in critically ill patients [1,2]. Its mortality rate is higher than those of bloodstream infections caused by Gram-positive and Gram-negative bacteria in intensive care units (ICU) [3]. While the US has one of the lowest crude mortality rates (~25%) among developed countries, Brazil has one of the highest (≥50%) among developing ones [4,5]. The healthcare-associated factors for ICU candidemia include hemodialysis, mechanical ventilation, and central venous catheter (CVC), mainly as access for total parenteral nutrition (TPN) [6]. Even though *Candida albicans* is still the most frequent *Candida* species isolated in blood cultures, non-*albicans Candida* (NAC) species have emerged in many countries worldwide, mostly in developing ones [5]. Among NAC species, *C. glabrata* is the most common in the US followed by *C. parapsilosis*, the most common in Brazil [4,5]. In contrast to the prior colonization-dependent vertical transmission of *C. albicans*, the horizontal transmission of *C. parapsilosis* is supported by its propensity to colonize the hands of healthcare workers, to form biofilm on CVC, and to grow rapidly in TPN, making it a *Candida* species of high concern in ICUs [7,8]. Even more worrisome are the increasingly frequent candidemia outbreaks caused by *C. parapsilosis* resistant to fluconazole (FLC) [9,10,11,12,13], the main antifungal drug used for candidemia treatment in developing countries [5].

The coronavirus disease 2019 (COVID-19) pandemic has required an unprecedented increase in critical care capacity even in developed countries, and ICUs have become overwhelmed despite surge strategies [14]. Taking into account that acute respiratory distress syndrome and acute kidney injury are two of the most common complications in COVID-19 patients admitted to ICUs, requiring mechanical ventilation and hemodialysis [14], those patients are in a high-risk group for candidemia [15,16]. On the other hand, the strict adherence to hand hygiene and disinfection practices recommended to minimize the risk of nosocomial cross-contamination or transmission of severe acute respiratory syndrome coronavirus 2 (SARS-CoV-2) during the COVID-19 pandemic [17] might also contribute to minimize the horizontal transmission of *C. parapsilosis* in critical care setting.

Throughout 2019, a large candidemia outbreak caused by FLC-resistant *C. parapsilosis* (FRCP) with Erg11-Y132F mutation, associated with a high 30-day crude mortality rate, occurred in the ICU from a Brazilian adult cancer referral center. FRCP isolates from blood cultures, pericatheter skins, healthcare workers’ hands, and nosocomial surfaces were genetically identical by microsatellite analysis, strongly supporting the horizontal transmission of clonal isolates. Despite two attempts at environmental decontamination in late 2019, FRCP candidemia episodes remained a persistent and increasing challenge in early 2020 [18]. Since the first COVID-19 case in Brazil was reported on 25 February 2020 [19], many preventive measures, such as proper hand hygiene, were reinforced to minimize SARS-CoV-2 contamination in Brazilian hospitals [20]. However, in May 2020, an unexpected increase in the incidence of FRCP candidemia was reported in a Brazilian cardiology referral center, which provides emergency cardiac care for inpatients from the cancer center, suggesting an inter-hospital transmission of FCRP isolates through shared ICU patients. Therefore, the main aim of this work was to investigate an inter-hospital candidemia outbreak caused by FRCP isolates during the first year of the COVID-19 pandemic in Brazil. Worryingly, most of the FRCP isolates from the cardiology center presented the same genetic profile and Erg11 alterations of the strain that has been causing the persistent outbreak in the cancer center. This highlights the spread of clonal isolates from the Brazilian cancer referral center to the cardiology one and indicates that the improved infection prevention and control practices during the first year of the COVID-19 pandemic in Brazil were not effective at all in interrupting the horizontal transmission of this emerging FRCP strain.

## 2. Materials and Methods

**Figure 1:**
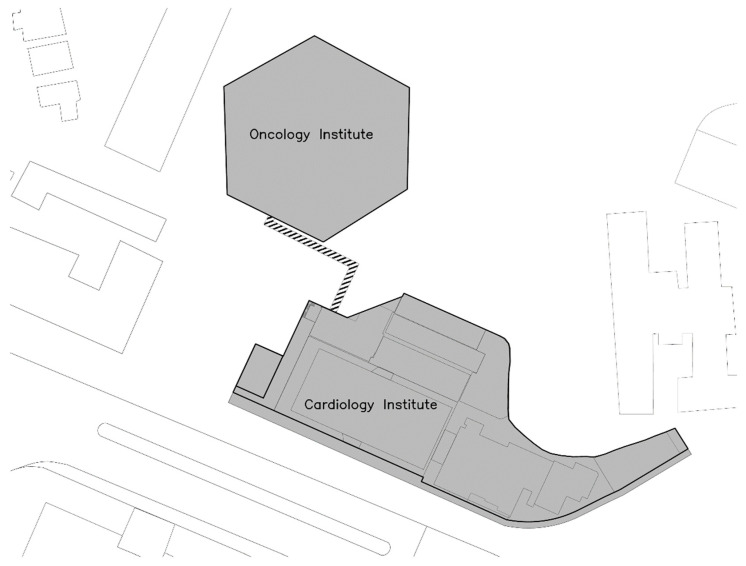
Physical connection between the independent buildings of the Brazilian referral hospitals.

The study analyzed *C. parapsilosis* bloodstream isolates obtained in 2020 from two Brazilian medical referral centers—an oncology institute and a cardiology institute. Although these hospitals are independent buildings, they present a physical connection for the transit of employees and patients (Figure 1). Both institutes are a 500-bed centers, and 12 inpatients were transferred from the oncology hospital to the cardiology one in 2020.

### 2.1. Species Identification and Fluconazole Susceptibility Testing

*Candida* bloodstream isolates obtained from the Brazilian cancer and cardiology centers in 2020 were submitted to species identification by matrix-assisted laser desorption ionization time-of-flight mass spectrometry (Vitek MS, bioMérieux, Marcy-l’Étoile, France), and to FLC susceptibility testing according to Clinical and Laboratory Standards Institute guideline M44 [21].

### 2.2. Multilocus Microsatellite Typing

All *C. parapsilosis* sensu stricto isolates recovered from distinct candidemia episodes in both medical centers in 2020 were genotyped by microsatellite analysis through PCR amplification of eight different loci [22], and compared to the FRCP genotype responsible for the outbreak in the cancer center in 2019 and the reference strain, ATCC 22019 [18]. After separating on 3% agarose gel and staining with GelRed™ (Biotium, Fremont, CA, USA), PCR products were submitted to the UVITEC gel documentation system (Cleaver Scientific, Rugby, Warks, UK) for visualization. Bionumerics software v. 7.6 (Applied Maths, Sint-Martens-Latem, Belgium) was employed to evaluate the similarity of allelic profiles and the clustering, by the Dice coefficient and unweighted pair group method with arithmetic mean, respectively. A cluster was defined as a group of two or more isolates with identical allelic profiles [23].

### 2.3. ERG11 Gene Sequencing

PCR amplification and sequencing of the *ERG11* gene of all *C. parapsilosis* isolates from the cardiology center were carried out with four specific primers [24]. PCR products were purified by ExoSAP-IT™ (Thermo Fisher Scientific, Waltham, MA, USA) and sequenced with 3500 Genetic Analyzer (Applied Biosystems, Foster City, CA, USA). MEGA v. X [25] was employed to analyze and compare the *ERG11* sequences with the corresponding sequences of *C. parapsilosis* ATCC 22019 (GenBank accession no. GQ302972) and FRCP genotype responsible for the outbreak in the cancer center in 2019 [18].

## 3. Results

**Figure 2:**
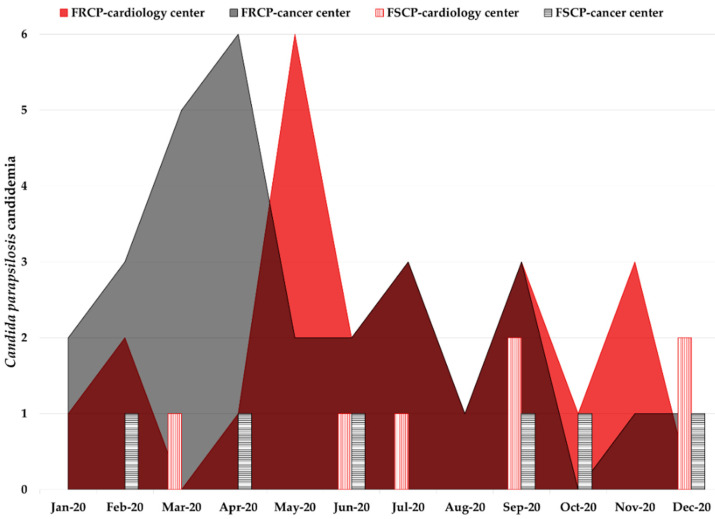
Sixty-five candidemia episodes caused by fluconazole-resistant *C. parapsilosis* (FRCP) or fluconazole-susceptible *C. parapsilosis* (FSCP) in the Brazilian cancer (*n* = 35) and cardiology (*n* = 30) centers in 2020. Colorful areas indicate FRCP candidemia cases and bars indicate FSCP cases.

**Figure 3:**
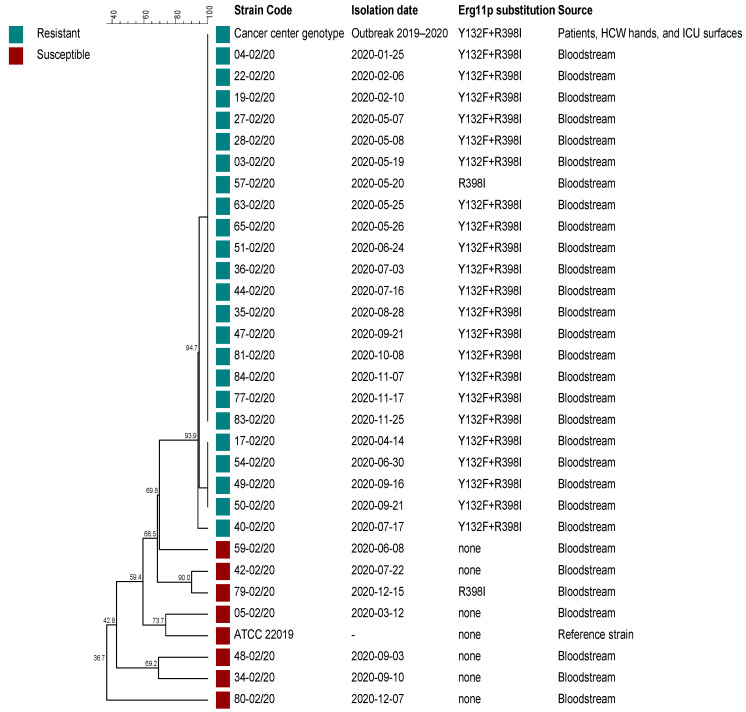
Dendrogram showing the clustering, the fluconazole susceptibility profile (resistant or susceptible), and Erg11p substitutions of *Candida parapsilosis* sensu stricto isolates obtained from the Brazilian cardiology center in 2020, the cancer center genotype [18] and the ATCC 22019 strain. HCW = healthcare workers, ICU = intensive care unit.

In March 2020, a series of modifications were implemented in the infection control practices at both the cancer and cardiology centers with the advent of the COVID-19 pandemic, aiming at controlling the nosocomial spread of SARS-CoV-2. Those procedures included, among several others, the mandatory wear of surgery masks, as well as regular hands hygiene and surface decontamination with 70% alcohol. In parallel, it was also expected that the spread of other nosocomial pathogens would be attenuated. However, both Brazilian medical centers faced a persistent FRCP outbreak during 2020 (Figure 2). In the cancer center, the FLC resistance rate in *C. parapsilosis* candidemia slightly decreased from 90% (63 resistant isolates out of 70 isolates) in 2019 to 82.9% (29/35) in 2020, despite the 50% decrease in the number of episodes. The FRCP genotype responsible for 81.4% (57/70) of candidemia cases during the outbreak in the cancer center in 2019 [18] was detected in 57.1% (20/35) of the episodes in the same hospital in 2020. The reduction in these candidemia indices paralleled the at least 50% reduction in the number of hospitalizations, surgeries, ICU patients, and activities, implemented by the direction of the cancer institute as a measure for the containment of the COVID-19 spread. On the other hand, the functioning of the cardiology center was not only kept unaltered during the pandemic, but it also started to receive many COVID-19 patients; the number of *C. parapsilosis* candidemia episodes and the FLC resistance rate increased, respectively, from 22 to 30 and from 50% to 76.7% in 2020 compared to 2019. Of note, the cancer center genotype responsible for the 2019 FRCP outbreak was detected in 60% (18/30) of candidemia episodes in the cardiology center in 2020 (Figure 3).

All FRCP isolates from the cardiology center presented at least one Erg11p substitution (Figure 3), as follows: one isolate had only R398I alteration, and 22 isolates had the same alterations (Y132F + R398I) detected in FRCP isolates obtained from the cancer center in 2019 [18]. Only one of the seven fluconazole-susceptible *C. parapsilosis* isolates presented an Erg11p substitution (R398I).

## 4. Discussion

The emergence of FRCP candidemia cases in a Brazilian cardiology referral center that receives unstable cardiac patients from an adult cancer center under a large FRCP outbreak suggested an inter-hospital transmission of isolates through ICU patients sharing. Despite the recommended hand hygiene with alcohol-based hand sanitizer during the COVID-19 pandemic [26] being reinforced, as well as environmental decontamination, candidemia by FRCP isolates remained an ongoing challenge in both hospitals throughout 2020 (Figure 2). The cancer center reduced the number of hospitalizations, including those in the ICU, and surgeries to prevent nosocomial SARS-CoV-2 transmission. Such measures could explain the considerable decrease in *C. parapsilosis* candidemia cases from 2019 (*n* = 70) to 2020 (*n* = 35). However, the cardiology center not only maintained its regular activities but also received COVID-19 patients, which may have contributed to the increase in *C. parapsilosis* candidemia cases from 2019 (*n* = 22) to 2020 (*n* = 30). Unprecedentedly, 60% of *C. parapsilosis* candidemia episodes in the cardiology center in 2020 were caused by the same FRCP genotype harboring Erg11-Y132F mutation from the cancer center (Figure 3), where it has been causing a persistent outbreak since 2019, as reported elsewhere [18]. The ineffectiveness of improved infection prevention and control practices in mitigating the horizontal transmission of this genotype makes its potential for spread disturbing.

*Candida parapsilosis* is the most common *Candida* species isolated from healthcare workers’ hands and ICU surfaces [27,28]. The emergence of FRCP strains on these sources is a major threat to critically ill patients who are often empirically treated with FLC for candidemia [18]. Previous studies have demonstrated the efficacy of alcohol-based disinfectants in eradicating *C. parapsilosis* from catheter hub and peritoneal dialysis bag medication port [29,30]. However, the uninterrupted horizontal transmission of the same FRCP genotype causing persistent candidemia outbreaks in our hospitals during the COVID-19 pandemic suggests this strain is resistant to alcohol-based hand sanitizers. Furthermore, the decontamination attempts with quaternary ammonium have not been able to cease FRCP transmission in the cancer center [18]. Indeed, quaternary ammonium disinfectants have shown no efficacy against *Candida* isolates [31]. Even more troubling, *C. parapsilosis* isolates have presented low susceptibility to some chlorine- or iodine-based disinfectants [31,32], and can survive on plastic healthcare surfaces longer than *C. auris* [33], an emerging multidrug-resistant *Candida* species in COVID-19 patients [15,34]. Therefore, our results warrant the urgency of investigations on new infection control measures for preventing the nosocomial transmission of FRCP isolates.

Although the global FLC resistance rates in *C. parapsilosis* range between 2 and 5% [8], much higher rates (15.1–90%) have been recently reported in some countries from Europe, Asia, Africa, and Latin America [18,35,36,37]. Molecular mechanisms combinations, including the overexpression of efflux pumps and point mutations in *ERG11* gene, may be involved in *C. parapsilosis* FLC resistance [24]. While R398I has been considered only a compensatory Erg11 alteration, Y132F is the main one detected in FRCP isolates and seems to confer potential for dissemination [35]. Moreover, it has been associated with high mortality rates [13,18]. Thus, the inter-hospital spread of FRCP clonal isolates with Erg11-Y132F during the COVID-19 pandemic makes this pathogen an even more evident emerging public health problem.

## Data Availability

*ERG11* sequences from fluconazole-resistant and fluconazole-susceptible isolates of the Brazilian cardiology center are available at GenBank (https://www.ncbi.nlm.nih.gov/genbank/, submitted on 8 November 2021 and 15 December 2021) under the accession numbers OL415514–OL415536 and OL907209–OL907215, respectively.
